# Management of renal tumors during pregnancy: case reports

**DOI:** 10.1186/s12882-021-02318-w

**Published:** 2021-04-09

**Authors:** Yi Zhao, Ziyi Yang, Weifeng Xu, Zhigang Ji, Jie Dong

**Affiliations:** 1 Department of Urology, Peking Union Medical College Hospital, Chinese Academy of Science, Beijing, China; 2 Peking Union Medical College Hospital, Peking Union Medical College, Chinese Academy of Medical Sciences, Beijing, China

**Keywords:** Pregnancy, Renal tumors, Renal cell carcinoma, Renal angiomyolipoma, Nephrectomy, Laparoscopy

## Abstract

**Background:**

Renal tumors during pregnancy are rare and the treatment requires evaluation of both the patient and the fetus. No consensus or guidelines has been proposed or verified in this field. We successfully managed three renal tumor cases during pregnancy and reviewed the relative literature.

**Case presentation:**

In the first renal cell carcinoma case diagnosed in the 21st week of pregnancy, laparoscopic retroperitoneoscopic partial nephrectomy was performed in the 26th week of pregnancy. In the second renal cell carcinoma case diagnosed in the 3rd week of pregnancy, laparoscopic retroperitoneoscopic radical nephrectomy was carried out after the abortion. In the third angiomyolipoma case who was diagnosed before pregnancy but received no treatment, we performed laparoscopic retroperitoneoscopic partial nephrectomy during the 17th week of pregnancy due to the rapid enlargement of the tumor.

**Conclusion:**

Although no consensus or guidelines for the management of renal tumors in pregnant patients has been proposed or verified, the general rules of kidney tumor management in non-pregnant patients and the guidelines for surgery in pregnancy could be referred to. Renal tumors found in pregnant patients require an individualized treatment regimen involving surgical timing, routes, techniques, and excision ranges, which should be decided by both the patients and the surgical teams.

## Background

Previous publications have reported the estimated incidence of cancer diagnosed during pregnancy is 1 in 1000 pregnancies, among which the most common ones are breast cancer, melanoma, cervical cancer, and lymphomas [1]. Urological cancers occur extremely rarely during gestation, approximately 13 in 1,000,000 pregnancies [2]. Renal cell carcinoma (RCC) is the most commonly reported renal neoplasms during pregnancy, followed by angiomyolipoma and blastoma [3]. In pregnant women with kidney tumors, issues that need to be considered include the safety of imaging, the effect of treatment on the fetus and the mother, the timing of intervention, and prognosis. These issues should be decided based on the assessment of the oncologic risk and renal function, the general health and comorbidities, and more importantly, the experience of the surgical team. However, there is no consensus or guidelines verified for the treatment of kidney tumors in pregnant patients. In this case report, we describe three successful management cases of pregnant patients who were diagnosed with kidney tumors and discuss our cases in the context of the relevant literature.

## Case presentation

Three renal tumor cases managed during pregnancy were reported. Our patients’ demographics, clinical presentation, tumor size and location, management as well as pathological data are presented in Table 1 and we described the details respectively.

**Table 1:** clinical features of the three clinical cases

Cases	Age	Presentations	Tumor size	Time of diagnosis	Time of surgery	Surgical approach	TNM staging	Pathological diagnosis
1#	36	No symptoms	7.9 × 6.9 × 6.2*cm*	21st week of pregnancy	26th week of pregnancy	Left retroperitoneoscopic NSS	T2aN0M0	RCC
2#	39	No symptoms	4.6 × 4.4 × 5.3*cm*	3rd week of pregnancy	After abortion	Right retroperitoneoscopic RN	T1bN0M0	RCC
3#	30	Left lower quadrant discomfort	25 × 13*cm*	One year before pregnancy	17th week of pregnancy	Left retroperitoneoscopic NSS	NA	renal angiomyolipoma

*RN* Radical nephrectomy, *NSS* Nephron-sparing surgery, *RCC* Renal cell carcinoma, *NA* Not applicable

### Case 1#

A 36-year-old female, Gravida 2, Para 1, at 21 weeks of pregnancy without remarkable history was incidentally found to have a 7.9 × 6.9 × 6.2*cm* mixed echoic signal mass with central vascularity in the middle of the left kidney on fetal ultrasound. Magnetic resonance imaging (MRI) was then performed and found the mass to be mixed intensity with multiple septa, suggesting a renal tumor and indicating there may exist bleeding and/or cystic change inside the tumor. No associated para-aortic lymphadenopathy or involvement of other organs was found. Referring to the current guidelines, radical nephrectomy is recommended for T2 (> 7 cm) tumors. However, some studies showed that partial nephrectomy for T2 or greater RCC may have a similar outcome to those of radical nephrectomy [4]. The patient had a strong aspiration to reserve the left kidney, and we had a rich experience in this field. After consultation with obstetricians and explained the perioperative risks, including surgery failure and tumor recurrence, the patient decided to take the operation. At 26 gestational weeks, a left retroperitoneoscopic partial nephrectomy was performed. After general anesthesia, the patient was placed in the right lateral position. After the establishment of the retroperitoneal space, four trocars were inserted on the left waist between the superior edge of the iliac spine and the inferior border of the rib. CO_2_ insufflation was commenced and maintained at 12 mmHg. The kidney was exposed by opening Gerota’s fascia. The renal artery was dissected and clipped by Bulldog for 28 min. The operative time was 100 min, with an estimated blood loss of 150 ml. During the operation, the patient’s blood pressure remained stable. Fetal cardiac activity was monitored throughout the procedure, and fetal stability was ascertained at the end of the procedure. A pathological examination after surgery showed RCC (WHO grade III) with CA9 (+), EMA (+), and Vimentin (+) in immunohistochemical stain (pT2aN0M0). The surgical margin was clear. The patient did not require any adjuvant treatment during the postoperative period and was discharged 6 days after the surgery. The creatinine level was 51 μmol/L (eGFR = 126 mL/min·1.73m^2^) 1 month after the surgery, compared to 47 μmol/L (eGFR = 138 mL/min·1.73m^2^) before surgery. Subsequently, the patient delivered a healthy full-term baby 12 weeks after the operation.

### Case 2#

A 39-year-old female, Gravida 1, Para 0, at 3 weeks of pregnancy with a history of anemia and multiple uterine leiomyomas was admitted into our hospital. A 4.6 × 4.4 × 5.3*cm* mass with increased vascularity in the middle of the right kidney was incidentally found by ultrasound. The contralateral kidney was normal. There was no evidence of the involvement of any other organ. The RENAL score was 8P. After consultation with obstetricians and discussion with the surgical team, the patient decided to terminate the pregnancy before the kidney surgery. Three weeks after the abortion, a right retroperitoneoscopic radical nephrectomy was carried out under general anesthesia. A trocar for the camera was introduced on the mid-axillary line, 2 cm above the iliac crest. The other two trocars for manipulation were introduced on the anterior and posterior axillary line below the coastal border respectively. CO_2_ insufflation was maintained at 15 mmHg. The operative time was 90 min with an estimated blood loss of 50 ml. The right adrenal gland was removed. Postoperative recovery was uneventful and the patient was discharged 6 days after the surgery. Pathology of the resected tumor showed renal clear cell carcinoma (WHO grade I) with cystic change and hemorrhage, without invasion into the renal capsule and or pelvis (pT1bN0M0). Immunohistochemical stain showed positive AE1/AE3. The creatinine level was 65 μmol/L (eGFR = 94 mL/min·1.73m^2^) 1 month after the surgery, compared to 64 μmol/L (eGFR = 95 mL/min·1.73m^2^) before surgery.

### Case 3#

A 30-year-old female, Gravida 1, Para 0, at 17 weeks of pregnancy presented with left lower quadrant discomfort. She was diagnosed with bilateral multiple renal masses a year before pregnancy without any treatment. However, the patient lost her primary radiological results and we could not find the detailed information of the tumor. Ten months later, the ultrasonography at 13 weeks of pregnancy showed the largest renal mass located in the lower pole of the left kidney, with the size of 16 × 14*cm*. As a result, we couldn’t ensure the tumor growth rate. She repeated the urologic ultrasonography at 17 weeks of pregnancy and the mass was shown to be 25 × 13*cm* with mixed echoic signals. We did a particular body examination after the patient was admitted. There were no skin characteristics of tuberous sclerosis, including hypopigmented macules, angiofibromas, and shagreen patches. No family history was reported. Therefore, we took no consideration of the diagnosis of tuberous sclerosis. After consultation with obstetricians and discussion with the patient, at 17 gestational weeks, a left retroperitoneoscopic partial nephrectomy was performed. After general anesthesia, the patient was placed in the right lateral position. A trocar for the camera was introduced on the mid-axillary line, 2 cm above the iliac crest. Two trocars were introduced on the anterior and posterior axillary line below the coastal border respectively. A third trocar was placed between them for instruments and manipulation. CO_2_ insufflation was maintained at 15 mmHg. The renal artery was dissected and clamped by Bulldog for 30 min. The operative time was 110 min, with an estimated blood loss of 100 ml. The blood pressure of the patient and fetal cardiac activity was stable throughout the procedure. On the day of surgery and 2 days after the surgery, the patient was treated with 40 mg/day intramuscular progesterone. Postoperative recovery was uneventful and the patient was discharged 6 days after the surgery. Pathological analysis revealed renal angiomyolipoma. The creatinine level was 42 μmol/L (eGFR = 163 mL/min·1.73m^2^) 1 month after the surgery, the same as that before surgery. The patient gave birth to a baby uneventfully at 39 weeks’ gestation.

## Discussion and conclusions

Although urological tumors diagnosed during pregnancy are rare, a timely diagnosis has prognostic benefits because the intervention would be much easier before the tumor progresses further and when the uterine is relatively small. Approximately 109 cases of renal malignancies have been reported so far, of which 91 are RCC [2]. The mainly reported symptoms include pain (50%), hematuria (47%), hypertension (18%), and the classical triad of hematuria, pain, and palpable mass (26%) [5]. Most of the renal neoplasms in pregnant women are found incidentally during fetal ultrasonography without remarkable presentation. Only one of our three patients showed abdominal discomfort, whose tumor is much larger than the other two. Since the symptoms are barely perceptible, diagnostic imaging becomes crucial in early diagnosis. During the evaluation of tumor status, the acuity of staging and the radiation exposure to the fetus should be balanced. Computed tomography (CT) scan, as a commonly used technique to evaluate tumors in non-pregnancy patients, is not the first choice for pregnant patients. Whether there exists a safety threshold for radiation exposure during pregnancy is still controversial [2, 6]. Before more evidence come out, imaging methods involving radiation should try to be avoided during the whole process of pregnancy. As a routine examination during pregnancy, urological ultrasound has a sensitivity comparable to that of CT for exophytic masses larger than 3 cm, which makes it a great screening tool. However, ultrasonography during pregnancy usually focuses only on the pelvic region and fetal structures, which may result in the delay of diagnosis. Therefore, some studies recommend a total abdominal ultrasound be done at least once during pregnancy [7]. Once the lesion is detected, MRI should be used to further evaluate the tumor type and peripheral involvement. All of our cases were initially diagnosed via urological ultrasound, among which, one received MRI afterward to confirm the diagnosis, and one received the CT scan after abortion. The ultrasonography-guided biopsy is recommended by some studies, which believe the risks and complications are not more severe than in nonpregnant patients [7]. In our two RCC cases, the tumor features showed in the ultrasound and MRI highly indicated malignancy. The angiomyolipoma in the third case grew rapidly with the risk of rupture and hemorrhage. Therefore, we didn’t perform a biopsy because all three conditions had clear indications for timely intervention. In cases of indeterminate masses, biopsy should be considered.

Not all of the kidney tumors found during pregnancy need urgent intervention. The surgical decision should be made based on the condition of the fetus and the mother, the aggressiveness of cancer, and more importantly, the wishes of the pregnant women. If the surgery is unavoidable, the timing of treatment is critical. The general principle for localized RCC (stage I or II) is that surgical intervention should be avoided in the first trimester, whereas performing the surgery during the second trimester or after the fetal lung matures is usually recommended. However, for stage III or IV tumors found in any trimester, pregnancy termination is recommended and appropriate treatment of RCC should be the priority [7]. In our first case, stage II RCC was found in the second trimester so the surgery was performed immediately. In our second RCC case, although the tumor found in the early first trimester was in stage I, the patient wished not to delay the treatment, so pregnancy termination was performed first, followed by kidney surgery. As for angiomyolipoma, some experts raise the possibility that pregnancy-related hemodynamic changes might lead to hemorrhage from angiomyolipoma [8]. Some authors have suggested embolization of these lesions if they are detected before pregnancy [9]. In our third case, the angiomyolipoma was diagnosed before pregnancy but didn’t receive treatment. The rapid growth of the tumor during pregnancy made it an urgent clinical condition. Therefore, what we learned from the case is that intervention of angiomyolipoma found in child-bearing period women before pregnancy should be considered.

Radical nephrectomy and nephron-sparing surgery are essential treatments for the management of RCC. According to National Comprehensive Cancer Network (NCCN) guidelines, the general principle for RCC management is that in stage I disease, partial nephrectomy is recommended for T1a renal mass, while for T1b tumors, partial nephrectomy shows a similar outcome to that of radical surgery. The curative therapy for patients with stages II and III disease is radical nephrectomy. Partial nephrectomy can only be performed in patients with locally advanced tumors if technically feasible and clinically indicated [10]. In previous reports, some T1 and almost all of the T2 renal tumors in pregnant women were treated by radical nephrectomy to shorten operation time and reduce perioperative complications. In our first case, the patient with a T2a tumor received partial nephrectomy based on the tumor features and expert experience of surgeons, which proved nephron-sparing surgery was feasible in some T2 tumors. Although the patient in the second case had a T1b tumor, the surgical team decided to perform a radical nephrectomy because of the patient’s wishes. Angioembolization is an effective option in reducing tumor size for angiomyolipoma. Twenty-six pregnant women with angiomyolipoma were treated with an operation or/and embolization till 2015 [11]. It is suggested that contrast and radiography may lead to fetal anomaly or development. However, some studies showed that embolization can be performed after 12 weeks of gestation which had minimal fetal radiation exposure [12].

**Fig. 1:**
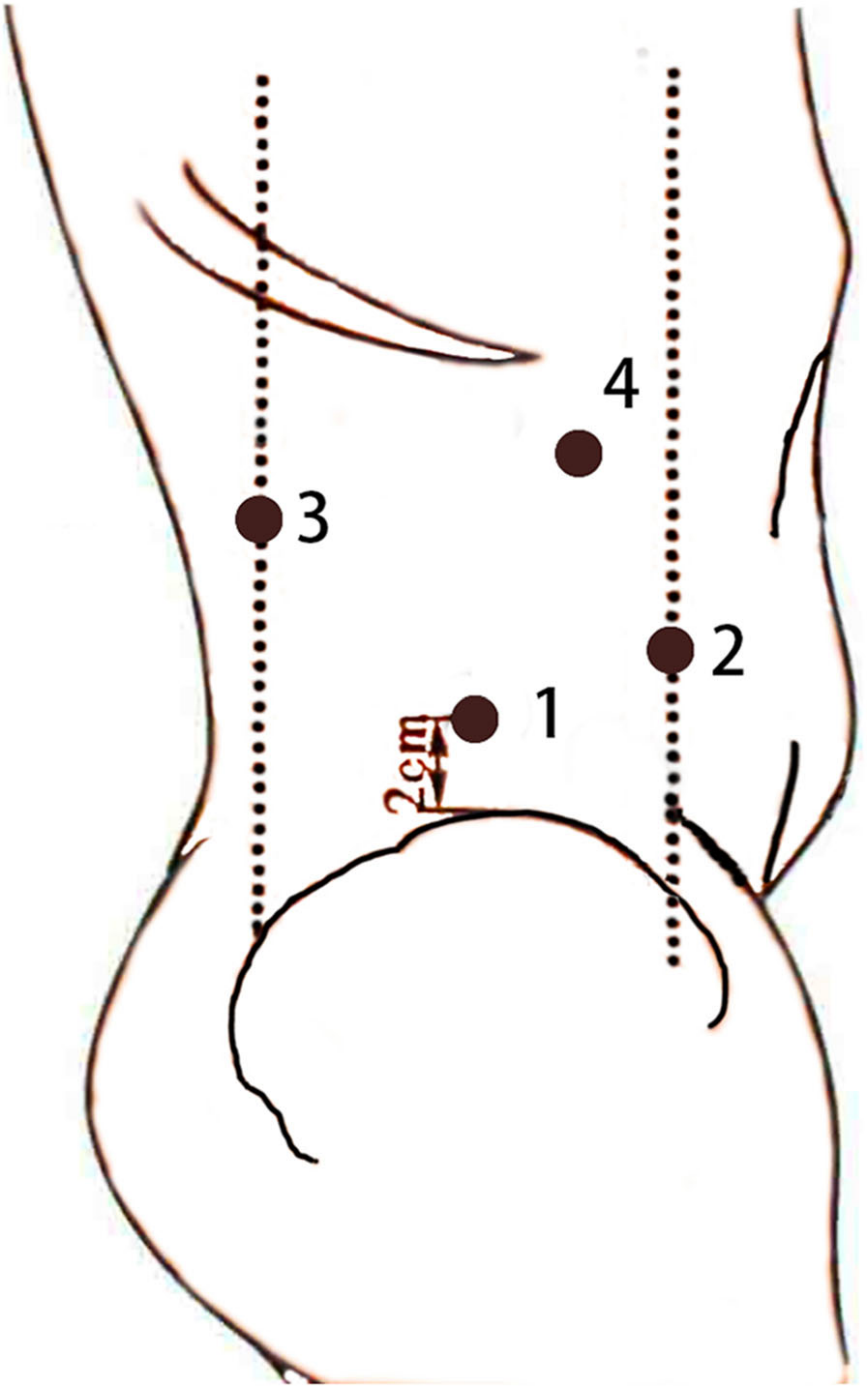
Trocar positions. Note that: #1 which on the mid-axillary line, 2 cm above the iliac crest meat camera port. #2 (anterior axillary line) and #3 (posterior axillary line) are the surgeon’s ports. #4 (between 2# and 3#) for instruments and manipulation is the assistant port

In recent years, with the development of surgical techniques and surgeons’ experience, laparoscopy has become gradually acceptable in pregnancy. An evidence-based guideline on laparoscopy in pregnancy commissioned by the British Society for Gynecological Endoscopy recommends laparoscopic surgery in pregnancy be carried out in settings where adequate time, laparoscopic expertise and monitoring facilities are available [13]. Laparoscopic surgery is associated with shorter surgical time and a faster recovery in both pregnant and non-pregnant patients. As for the transabdominal-transperitoneal route versus the lateral retroperitoneal route during pregnancy, surgery in the lateral position can effectively avoid the disturbance of the abdominal cavity caused by the operation and reduce the risk of uterine injury. Besides that, compared to the transperitoneal approach, the retroperitoneal approach can reduce the impact of abdominal pressure elevation and the acid-base balance disturbance caused by CO2 insufflation. These three cases all received laparoscopic surgery via lateral retroperitoneal route performed by laparoscopists with adequate skills, which improved the prognosis, minimized bleeding amount, and shortened the surgical time. The trocars’ positions of these three cases are shown in Fig. 1.

In the case of RCC during pregnancy, timely diagnosis and management are crucial in predicting maternal-fetal prognosis. The creatinine level and urological ultrasound of these three patients were followed 1 month after the surgery, which all showed no anomalies. Reoccurrence was not found in all three patients during long-term follow-up. The patients in case one and three both delivered a full-term healthy baby.

We also find some pregnant women who are diagnosed with renal angiomyolipoma are treated with conservative follow-up. A case was reported that a pregnant woman with a 10 cm mass suggestive of angiomyolipoma in the left kidney had a spontaneous rupture at the 20th week of her pregnancy, and the operation was performed until the 34th week [14]. It was reported that almost 75% of renal angiomyolipoma pregnant women had a risk of tumor rupture [11]. We did not find the literature on conservative management in renal cell carcinoma pregnant women.

In conclusion, although no consensus or guidelines for the management of renal tumors in pregnant patients has been proposed or verified, the general rules of kidney tumor management in non-pregnant patients and the guidelines for surgery in pregnancy could be referred to. Every pregnant patient needs an individualized treatment involving surgical timing, routes, techniques, and ranges based on the condition of the mother, the fetus, the tumor stages as well as the experience of the surgical team.

## Data Availability

All data generated or analyzed during this study are included in this published article.

## Abbreviations

RCC: Renal cell carcinoma
MRI: Magnetic resonance imaging
CT: Computed tomography
RN: Radical nephrectomy
NSS: Nephron-sparing surgery
NCCN: National Comprehensive Cancer Network
NA: Not applicable
